# Near-infrared fluorophores methylene blue for targeted imaging of the stomach in intraoperative navigation

**DOI:** 10.3389/fbioe.2023.1172073

**Published:** 2023-04-13

**Authors:** Zhidong Wang, Lin Mei, Xiao Yang, Tiantian Jiang, Tingkai Sun, Yuanhao Su, Youshen Wu, Yuanyuan Ji

**Affiliations:** ^1^ Department of General Surgery, The Second Affiliated Hospital, Xi’an Jiaotong University, Xi’an, China; ^2^ Scientific Research Center and Precision Medical Institute, The Second Affiliated Hospital, Xi’an Jiaotong University, Xi’an, China; ^3^ School of Chemistry, Xi’an Jiaotong University, Xi’an, China

**Keywords:** methylene blue (MB), near-infrared (NIR) fluorescence imaging, gastric tissues, gastric tumors, intraoperative navigation

## Abstract

Near-infrared (NIR) fluorescence imaging-guided surgery is increasingly concerned in gastrointestinal surgery because it can potentially improve clinical outcomes. This new technique can provide intraoperative image guidance for surgical margin evaluation and help surgeons examine residual lesions and small tumors during surgery. NIR fluorophores methylene blue (MB) is a promising fluorescent probe because of its safety and intraoperative imaging in the clinic. However, whether MB possesses the potential to perform intraoperative navigation of the stomach and gastric tumors needs to be further explored. Therefore, the current study mainly validated MB’s usefulness in animal models’ intraoperative imaging of stomach and gastric tumors. NIR fluorophores MB can exhibit specific uptake by the gastric epithelial cells and cancer cells. It is primarily found that MB can directly target the stomach in mice. Interestingly, MB was applied for the NIR imaging of gastric cancer cell xenografts, suggesting that MB cannot specifically target subcutaneous and orthotopic gastric tumors in xenograft models. Thus, it can be concluded that MB has no inherent specificity for gastric tumors but specificity for gastric tissues. Apparently, MB-positive and negative NIR imaging are meaningful in targeting gastric tissues and tumors. MB is expected to represent a helpful NIR agent to secure precise resection margins during the gastrectomy and resection of gastric tumors.

## 1 Introduction

Near-infrared (NIR) fluorescence imaging is a potential technique to identify tumors and sentinel lymph nodes intraoperatively. In the clinic, the application of NIR fluorescence imaging together with the targeted contrast agents makes it possible for enormous advance in intraoperative image-guided surgery (Haque et al., 2017). Currently, methylene blue (MB), a US Food and Drug Administration (FDA)-approved medicine, has been clinically used in intraoperative image-guided surgery (Tanaka et al., 2009). For instance, as a contrast agent, MB could be utilized to examine the bile leaks during cholecystectomy *via* injecting directly into the gall bladder (Sari et al., 2005). Previous findings also reported that the NIR fluorophore MB could be employed for intraoperative identifying the pancreas in abdominal surgery (Winer et al., 2010). Moreover, by NIR fluorescence imaging, MB intraoperatively detects breast tumors (Tummers et al., 2014). Since researchers have thoroughly evaluated the safety of MB usage, MB may be available in the future.

MB is a heterocyclic aromatic compound, which makes an excitation peak of 670 nm and an emission peak of 690 nm (Selvam and Sarkar, 2017). MB serves as a visible wavelength non-specific fluorophore when a millimole dose is used, and a NIR 700 nm fluorophore when a micromole dose is used (Ashitate et al., 2012b). Traditionally, MB of high millimolar doses has been used to examine sentinel lymph nodes in melanoma under bright light (Peek et al., 2017). However, MB of low micromolar doses exhibits fluorescence properties with an emission peak of 690 nm (DiSanto and Wagner, 1972). At present, the characteristics and functions of MB as a NIR fluorophore are becoming the focus of attention.

Fluorescence navigation surgery has the potential clinical application in the vascular or lymphatic system and tumor, and it has the ability to evaluate surgical resection margins as well as the extension of a lesion, which could be considered as a new method in minimally invasive surgery (Namikawa et al., 2020). Undoubtedly, fluorescence navigation surgery is a future direction which could be applied widely in the medical field for intraoperative gastroendoscopy and gastric tumors (Namikawa et al., 2015). When performing gastrointestinal surgery, it is necessary to mark the target lesions before surgery if they are palpable (Leonards et al., 2017). Surgeons will benefit substantially from the capability of fluorescent imaging to merge into the traditional color images when NIR is used in real-time because of the excellent visualization and detection of invisible target lesions to the naked eye (Mahalingam et al., 2017). More importantly, with the help of the specific features of NIR fluorescence, surgeons can locate the exact places of fluorescent lesions associated with the surrounding structures (Wen et al., 2018).

**SCHEME 1:**
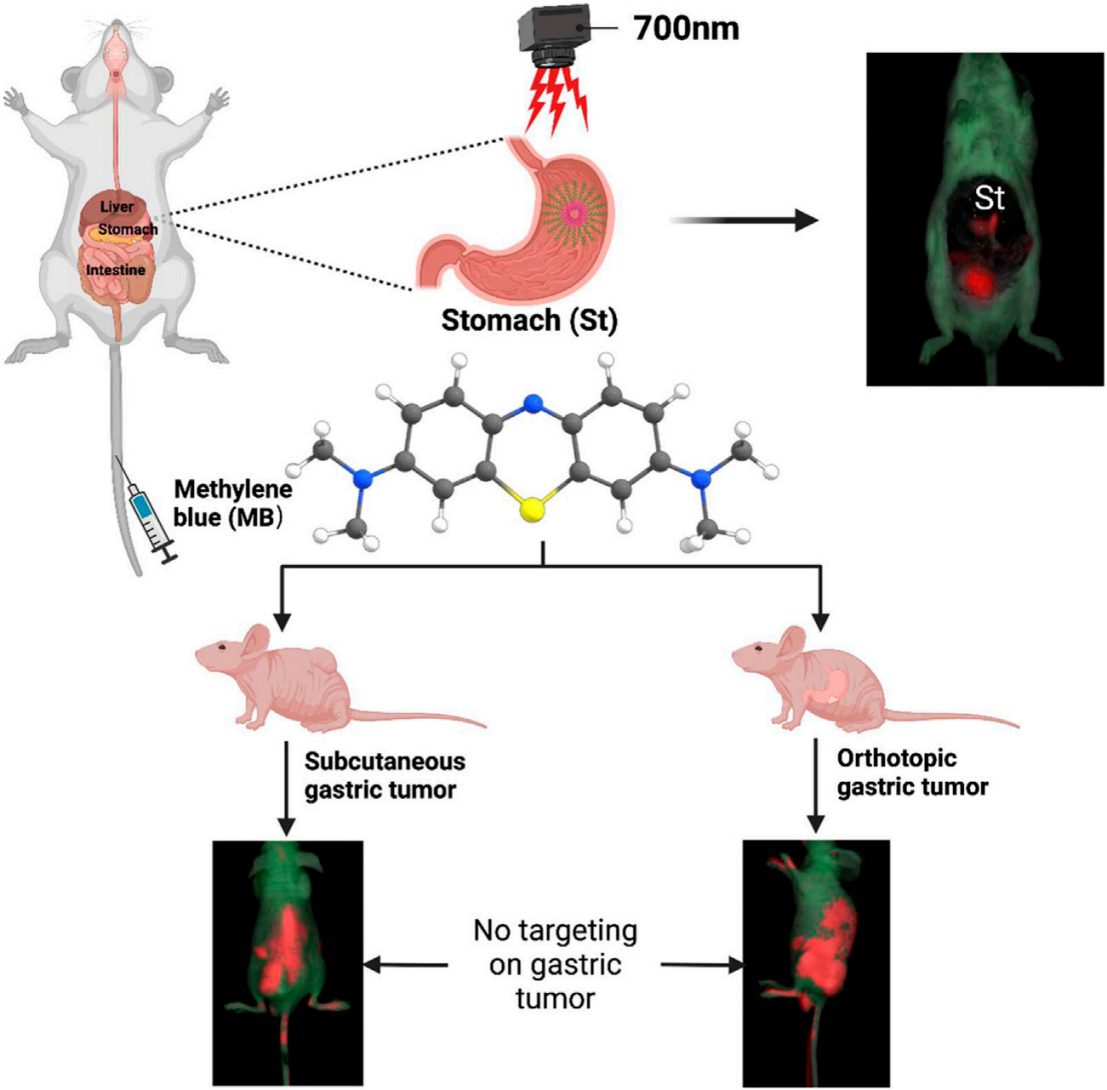
Schematic illustration of near-infrared (NIR) fluorophores methylene blue (MB) for targeted imaging of stomach rather than targeted imaging of gastric tumor.

Clinically, NIR fluorescent contrast agents targeting the stomach and gastric tumors are rare. On the other hand, MB is a promising fluorescent probe because of its safety, visualization, and intraoperative imaging (Liu et al., 2008). It has been found that MB is advantageous in some surgery systems which are guided by fluorescence images, including cholecystectomy and breast tumor (Sari et al., 2005; Tummers et al., 2014). However, whether MB possesses the potential to perform intraoperative navigation of the stomach and gastric tumors needs to be further explored. Therefore, the current study mainly validated MB’s usefulness in intraoperative imaging of stomach and gastric tumors in animal models. As illustrated in Scheme 1, NIR fluorescence signals of MB were observed for intraoperative NIR imaging of mice’s abdominal cavities, indicating that the NIR fluorophore MB targeting on the stomach in mice. Interestingly, NIR fluorescence signals of MB were not shown in the nude mice’s subcutaneous and orthotopic gastric tumors. MB-positive imaging was in the gastric tissues, and MB-negative imaging was in the gastric tumors. Thus, MB can be expected to secure precise resection margins during the gastrectomy and resection of gastric tumors in the future.

## 2 Materials and methods

### 2.1 Materials

Bovine serum albumin (BSA), methylthiazol tetrazolium (MTT), and methylene blue (MB) were bought from Sigma (St. Louis, MO, United States). HyClone™ RPMI 1640 Medium and dulbecco’s modified eagle medium (DMEM) was provided by Thermo Fisher (Thermo Fisher Scientific, Waltham, MA, United States), and fetal bovine serum (FBS) were supplied from Gibco BRL (Carlsbad, CA, United States). The NIR fluorescence imaging system was from IVIS Lumina XR Series III from PerkinElmer (CA, United States).

### 2.2 Measurement of optical properties

Absorbance and fluorescence of NIR fluorophore MB were detected using fiber optic HR 2000 (200–1,100 nm) spectrometers (Ocean Optics, Dunedin, FL). All optical measurements were made in BSA media. In addition, the NIR fluorescence imaging system evaluated MB’s optical properties and fluorescence intensity.

### 2.3 Cell culture and cell viability

Human gastric epithelial cell lines GES-1 and human gastric cancer cell lines BGC-823 were obtained from the Cell Bank of the Committee on Type Culture Collection of the Chinese Academy of Sciences (Shanghai, China). GES-1 and BGC-823 cells were cultured with DMEM, 10% FBS, and ampicillin/streptomycin at 37°C in a 5% CO_2_ humidified atmosphere and were added to RPMI 1640 Medium. Then GES-1 and BGC-823 cells were incubated with MB with different concentrations (0.01, 0.1, 1, 10, and 100 μM) for 60 min, and MTT assay was used to identify cell viability. Moreover, cell viability of GES-1 and BGC-823 cells was also observed with MB (1 μM) at various NIR irradiation exposure times (30, 60, 90, 120, and 150 min) *via* MTT assay.

### 2.4 Human gastric epithelial and gastric cancer cells labeled with MB

To analyze the imaging of MB to GES-1 and BGC-283 cells, we seeded the GES-1 and BGC-283 cells onto sterilized glass coverslips in 24-well plates treated with MB (5 µM), and then we observed the cellular fluorescence under the confocal microscope (Leica SP8, Germany). In addition, the fluorescence intensity of GES-1 and BGC-283 cells incubated with MB was conducted the analysis by using ImageJv1.8.0 (National Institutes of Health, Bethesda, MD).

### 2.5 *In vivo* biodistribution and clearance of NIR fluorophores MB

The Department of Experimental Animals, Health Science Center, Xi’an Jiaotong University supplied the CD-1 male mice (6-week-old). After we injected MB (250 µM/100 µL) in saline containing 10% BSA intravenously into CD-1 mice, we found that the NIR fluorescence signals were at 4 h post-injection under the NIR fluorescence imaging system for the biodistribution and clearance of MB *in vivo*. After finishing the imaging, we euthanized mice and dissected major organs for fluorescence imaging. Each sample used three mice.

### 2.6 Establishment of gastric tumor model and intraoperative imaging

All animal experiments were performed according to the Guidelines for Use and Care of Animals at Xi’an Jiaotong University (No. 2019–293). The Department of Experimental Animals, Health Science Center, Xi’an Jiaotong University supplied male nude mice (8-week-old). To establish a subcutaneous gastric tumor model, 1 × 10^7^ BGC-823 cells suspended in 200 μL of saline/Matrigel were inoculated subcutaneously at the left flank of the male nude mice. Furthermore, to establish an orthotopic gastric tumor model, we maintained the nude mice under anesthesia and opened the abdomen of the nude mice, and then injected 1 × 10^7^ BGC-823 cells suspended in 100 μL of saline/Matrigel directly between the submucosa and the muscular layer of the stomach, finally sutured the wound according to the previous method (Kang et al., 2021). When the long axis of the tumor diameter arrived at 1–2 cm or palpation of abdominal nodules, MB (250 µM/100 µL) in saline containing 10% BSA was injected into tumor-bearing mice through the tail vein. In brief, isoflurane was used to anesthetize the tumor-bearing mice with the image of 0.5, 1, 2, 4, 8, 24, 48, and 72 h under NIR imaging (PerkinElmer IVIS Lumina XR Series III, United States). After finishing the real-time imaging, we euthanized mice and dissected major organs and gastric tumor tissues for fluorescence imaging. Each sample used three mice.

### 2.7 Fluorescence imaging and hematoxylin and eosin (H&E) staining

Excised organs and gastric tumor tissues were trimmed and embedded in the OCT compound. Then, a cryostat (Leica, Germany) cut the frozen sections whose thickness was 10 μm. Next, the fluorescence imaging was displayed on the confocal microscope (Leica SP8, Germany), but H&E stained one part of the serial sections. Finally, the image of the sectioned slides was generated using a Nikon 50i microscope (×20 objective or ×10 objective).

### 2.8 Quantitative analysis of fluorescence images

ImageJ v1.8.0 (National Institutes of Health, Bethesda, MD) was used to quantify the fluorescence and background intensities of a region of interest over each tissue. The following details demonstrated the calculation of the signal-to-background ratio (SBR) (Ji et al., 2019):

$$SBR=\frac{I_{ROI}}{I_{Auto}}$$,

where the average intensity of an ROI is represented by $I_{ROI}$ and the intensity of the muscle was denoted by $I_{Auto}$ . The same formula was applied to calculate the tumor-to-background ratio (TBR), and $I_{T}$ represented the intensity of the tumor tissue.

$$TBR=\frac{I_{T}}{I_{Auto}}$$

### 2.9 Statistical analysis

The data were analyzed using Prism 8 software (GraphPad, San Diego, CA). Results were illustrated as the mean ± SEM for all the images on the NIR imaging system and confocal microscope analyses.

## 3 Results and discussion

### 3.1 Structural characteristics and optical properties of NIR fluorophores MB

**FIGURE 1:**
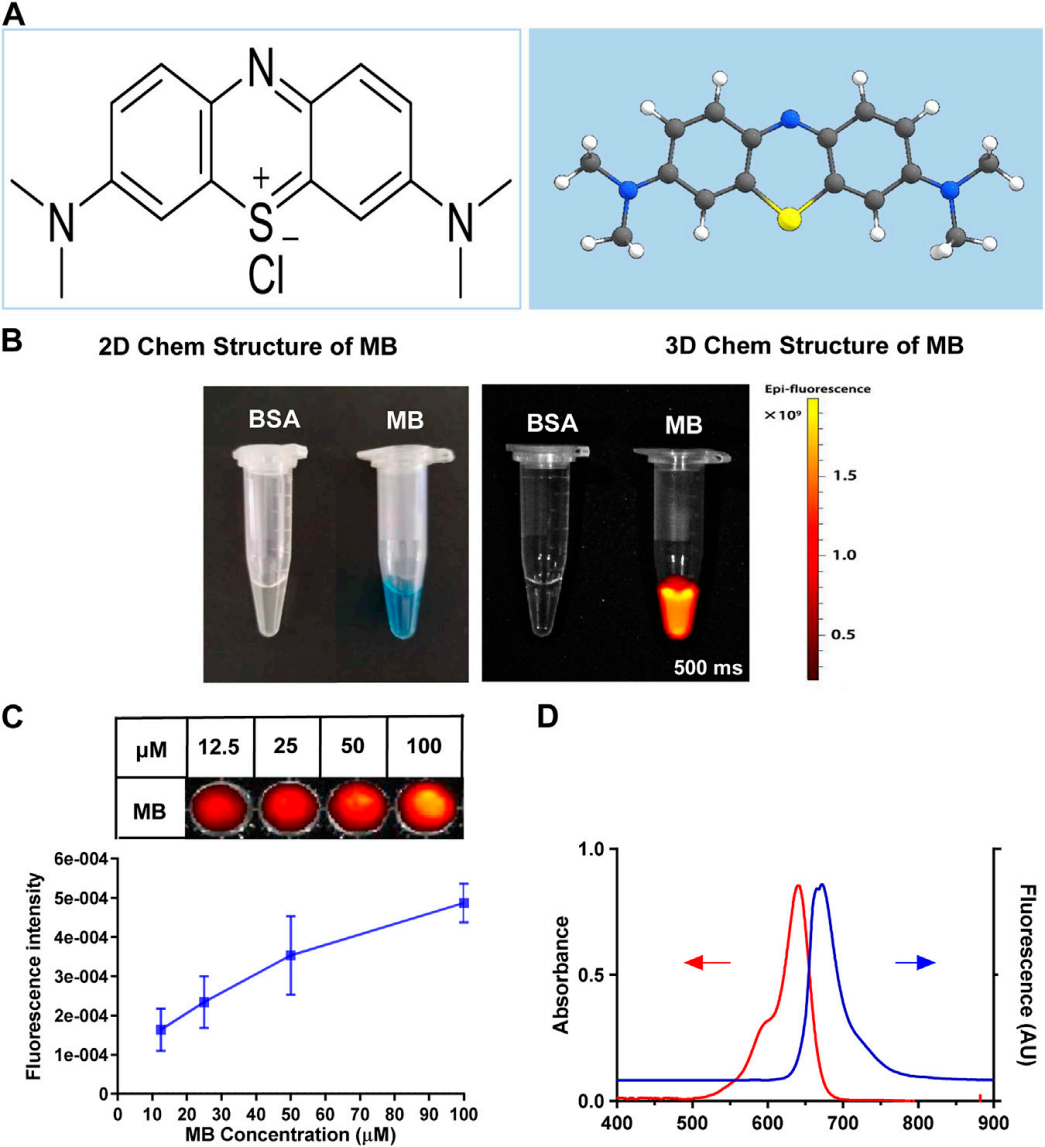
Structural characteristics and optical properties of MB. **(A)** 2D and 3D chemical structure of MB. **(B)** Optical properties of MB in 10% bovine serum albumin (BSA, 100 µM) were observed under the NIR fluorescence imaging system. **(C)** The fluorescent intensity of MB in 10% BSA (12.5, 25, 50, and 100 µM) was also examined by the NIR fluorescence imaging system. **(D)** Ocean Optics assays MB’s representative absorption and fluorescence spectra in 10% BSA (5 µM).

In Figure 1A, the 2D and 3D chemical structures of NIR fluorophores had been displayed. Compared with the bovine serum albumin (BSA), the NIR fluorescence of MB appeared to be more significant (Figure 1B). Additionally, the fluorescence intensity of MB can enhance with increasing fluorophore dose (12.5, 25, 50, and 100 µM) (Figure 1C). MB showed an excitation peak of about 700 nm in the NIR I window (Figure 1D). MB, a water-soluble dye, is approved for clinical use by the US FDA. In addition, MB can show an excitation peak of about 700 nm in the NIR I window (700–900 nm), permitting NIR imaging at a more significant penetration depth (Lwin et al., 2020). The present study revealed that BSA could enhance MB’s solubility and fluorescence signals. Due to its good biocompatibility and high stability, BSA is currently used as a drug delivery vehicle, including cancer diagnosis and therapy (Ashkenazi, 2010).

### 3.2 Cell viability and fluorescence imaging of NIR fluorophores MB

**FIGURE 2:**
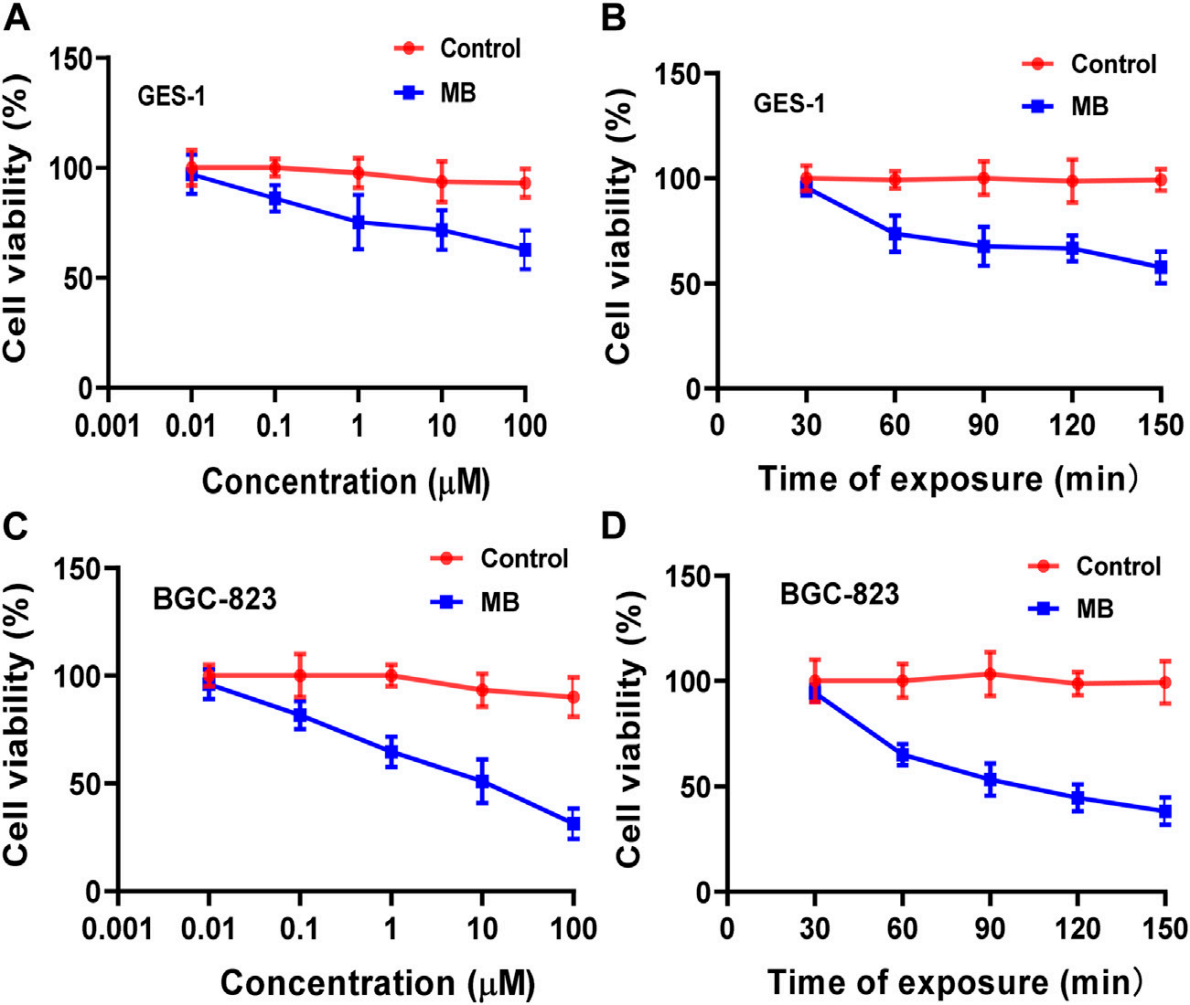
Cell viability of NIR fluorophores MB in human gastric epithelial GES-1 cells and human gastric cancer BGC-823 cells. **(A,C)** GES-1 and BGC-823 cells were incubated with MB with different concentrations for 60 min, and cell viability was determined compared to a control sample (human gastric epithelial and gastric cancer cells without MB). **(B,D)** Cell viability was also observed with MB (1 µM) at various NIR irradiation exposure times.

**FIGURE 3:**
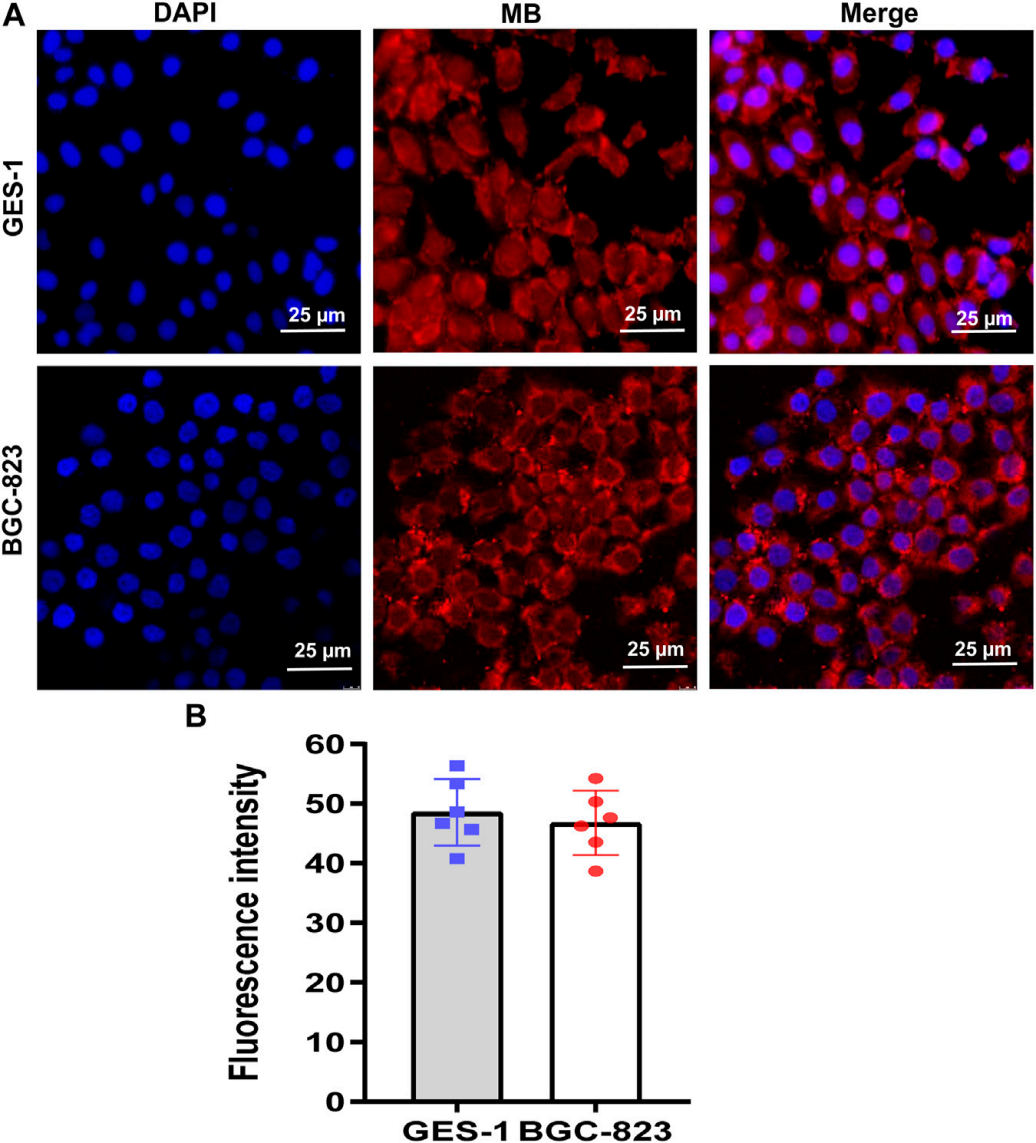
Fluorescence imaging of NIR fluorophores MB in human gastric epithelial GES-1 cells and human gastric cancer BGC-823 cells. **(A)** Representative image for DAPI (blue) and NIR (MB, red) in GES-1 and BGC-823 cells under the confocal microscope. **(B)** Fluorescence intensity of MB in GES-1 and BGC-823 cells were analyzed by ImageJ. Scale bars = 25 μm. Error bars show mean ± SEM.

The previous study demonstrates that MB can reduce the growth and viability of human pancreatic cancer Hs766T cells in a time- and dose-dependent way (Shen et al., 2021). To detect the cell viability of NIR fluorophores, human gastric epithelial GES-1 cells and human gastric cancer BGC-823 cells were incubated with MB with a different concentration for 60 min, and cell viability decreased with the increase of fluorophore concentration (Figures 2A, C). Moreover, GES-1 and BGC-823 cells were incubated with MB (1 µM) at various NIR irradiation exposure times, and cell viability declined with the enhancement of exposure times (Figures 2B, D). The present study indicates that the cell viability of human gastric epithelial GES-1 cells and human gastric cancer BGC-823 cells decreased with the increased fluorophore concentration. And cell viability of GES-1 and BGC-823 cells declined with the enhancement of exposure times. Thus, it is necessary to choose suitable concentrations and exposure times of MB in practical application. Furthermore, MB has been recognized as a potential tracer with selective uptake in human melanoma cells (Biberoglu et al., 2022). In the present study, to evaluate whether the NIR fluorophores MB can specifically target human gastric epithelial cells and human gastric cancer cells, GES-1 and BGC-823 cells were labeled with MB. Figures 3A, B showed that the NIR fluorescence signals were displayed in GES-1 and BGC-823 cells, especially the nucleus and cytoplasm, which possessed significant fluorescence signals, indicating that MB can take up and accumulate in gastric epithelial cells and cancer cells. The present results imply that MB can play a vital role in the fluorescence label of gastric epithelial cells and cancer cells.

### 3.3 *In vivo* biodistribution and clearance of NIR fluorophores MB

**FIGURE 4:**
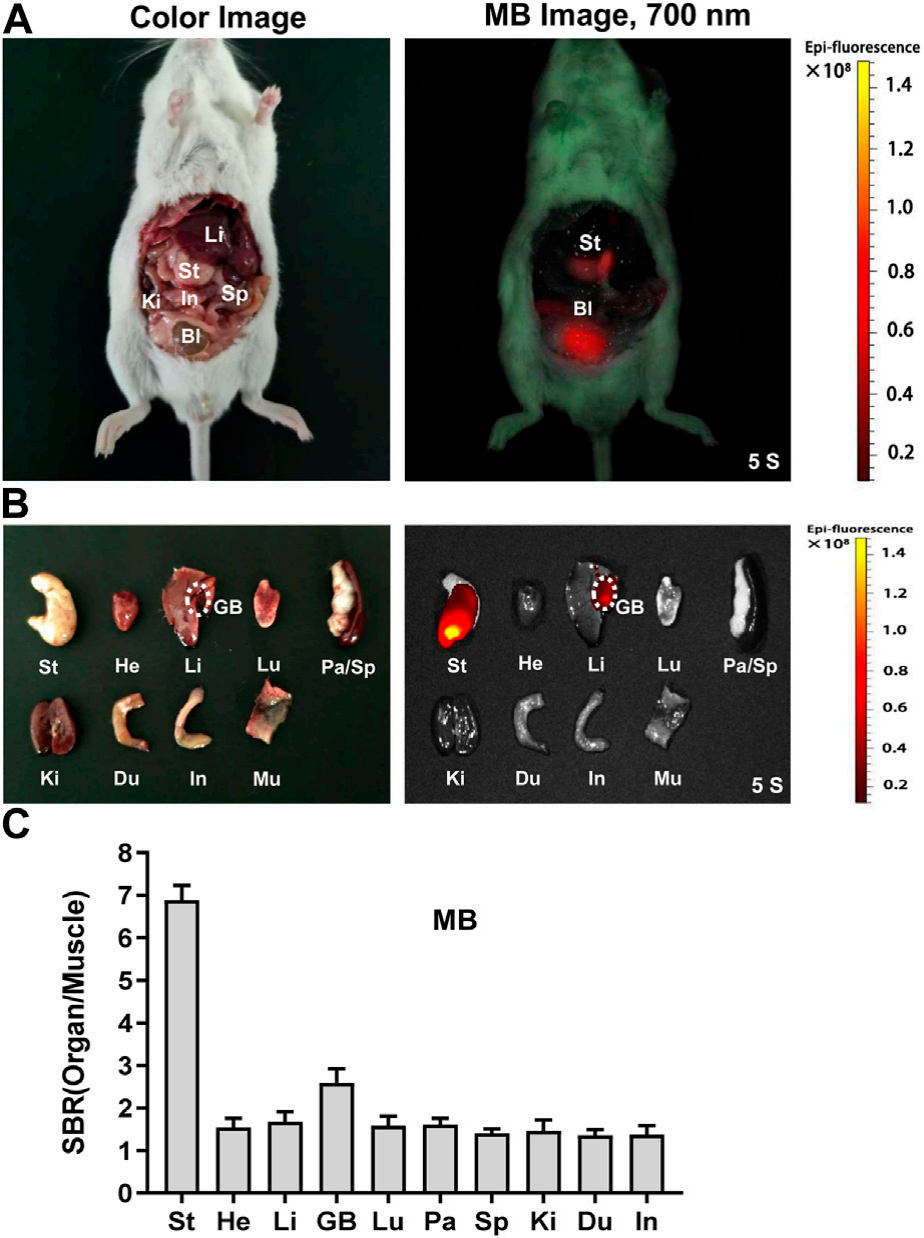
*In vivo* biodistribution and clearance of NIR fluorophores MB. MB was injected intravenously into CD-1 mice, and the NIR fluorescence signals were examined at 4 h post-injection under the NIR fluorescence imaging system. **(A)** NIR imaging of the abdominal cavity of mice. **(B)** NIR imaging of resected tissues and organs at 4 h. **(C)** The signal-to-background ratio (SBR) was calculated by comparing the signals of major organs against the surrounding muscle. Abbreviations are GB, gall bladder; He, heart; In, intestine; Ki, kidney; Li, liver; Lu, lung; Sp, spleen; St, stomach. Each sample used three mice.

Nowadays, five major areas of MB are available, including the fluorescence visualization of ureters, specific types of pancreatic tumors, breast cancer tumor margins, sentinel node biopsy of breast cancer, and parathyroid glands (Sobal et al., 2008). Due to its inexpensive and low toxicity, MB has been approved for clinical application (Cwalinski et al., 2020). However, MB targeting the stomach and gastric tumor is still unclear. As a result, the biodistribution and clearance of MB in the abdominal cavity of CD-1 mice were further studied. Intraoperative NIR imaging of the abdominal cavity of mice gave evidence of MB targeting the stomach in CD-1 mice at 4 h post-injection. The bladder and the stomach exhibited significant fluorescence signals after applying MB (Figure 4A). The NIR fluorescence signals of MB were detected mainly in the stomach, and the gall bladder displays weak signals. However, the fluorescence signals of the heart, lung, liver, spleen, and kidney were not revealed after the use of MB (Figure 4B). Consistently, resected organs’ signal-to-background ratio (SBR; organs vs. muscle) was remarkably high in the stomach (Figure 4C). The previous study reveals that MB can be cleared simultaneously by the kidney and liver, leading to high NIR fluorescence signals of the urine and bile (Wainwright and Crossley, 2002; Ashitate et al., 2012a). The current results support the previous reports and reveal exciting findings about MB-positive imaging in gastric tissue.

### 3.4 Real-time NIR targeted imaging in the subcutaneous gastric tumor

**FIGURE 5:**
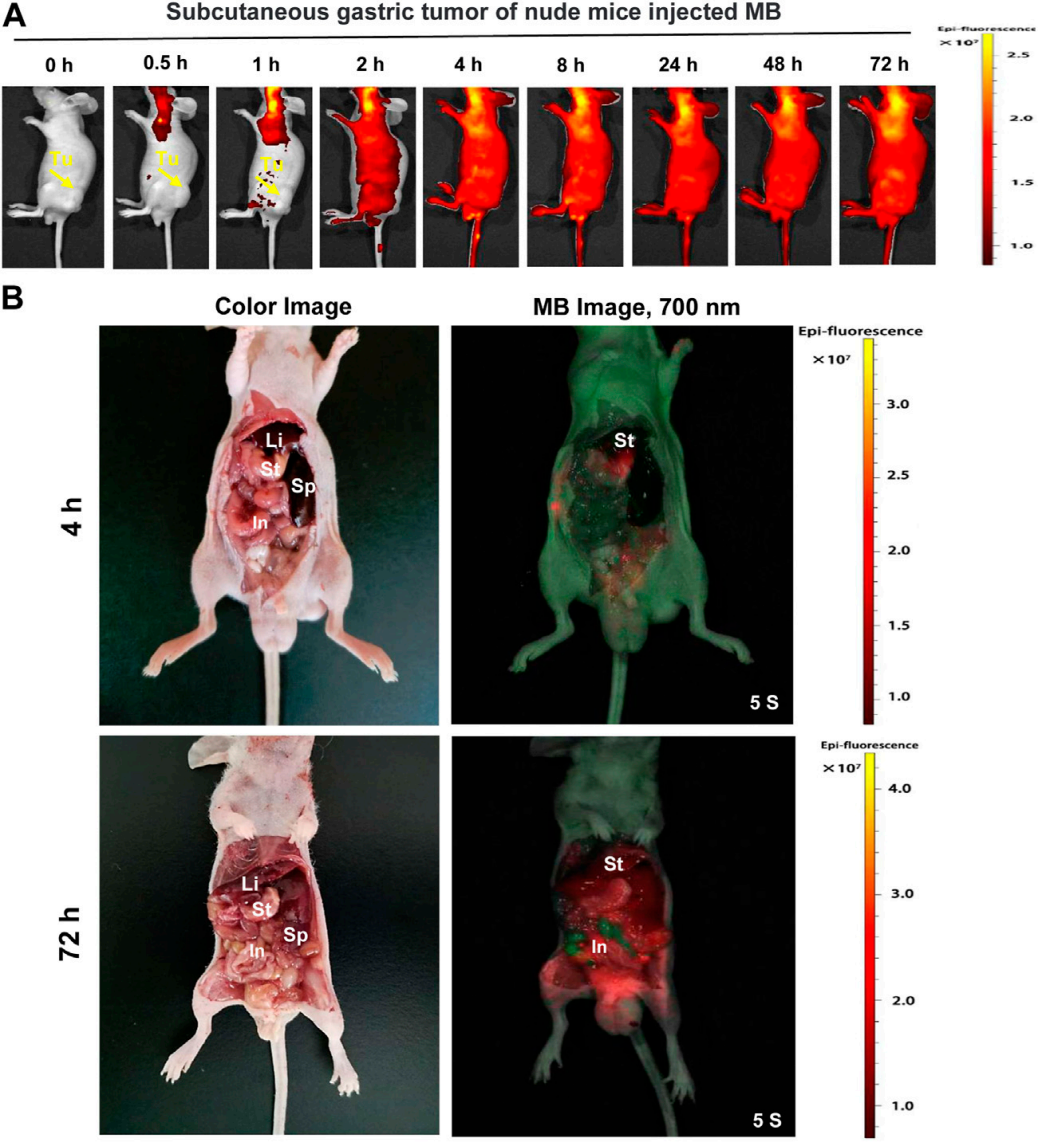
Real-time NIR targeted imaging after the injection of NIR fluorophores MB in the subcutaneous gastric tumor of nude mice. MB was injected intravenously into gastric subcutaneous tumor-bearing nude mice, and the NIR fluorescence signals were observed at 0.5, 1, 2, 4, 8, 24, 48, and 72 h post-injection under the NIR imaging system. **(A)** Real-time NIR fluorescence imaging of gastric subcutaneous tumor-bearing nude mice at different time points. **(B)** NIR imaging of the abdominal cavity of nude mice at 4 h and 72 h post-injection MB. Yellow arrows indicate tumor grafts in the left flank.

MB has been proposed to screen bladder and skin cancer (Yu et al., 1990; Malik et al., 2020). Furthermore, another study indicates that MB fluorescence imaging can identify breast cancer (Zhang et al., 2019). Therefore, we speculate on an exciting application of MB in gastric tumors detection with NIR fluorescence imaging. MB was injected intravenously into the nude mice’s subcutaneous gastric tumor and performed real-time intraoperative NIR imaging up to 72 h post-injection. Unfortunately, we cannot find the significant NIR fluorescence signals in the gastric tumor (Figure 5A). However, we observed strong NIR fluorescence signals in the stomach in the abdominal cavity of nude mice after 4 h and 72 h post-injection of MB (Figure 5B).

### 3.5 Organ and tumor specificity of MB at 4 h and 72 h post-injection

**FIGURE 6:**
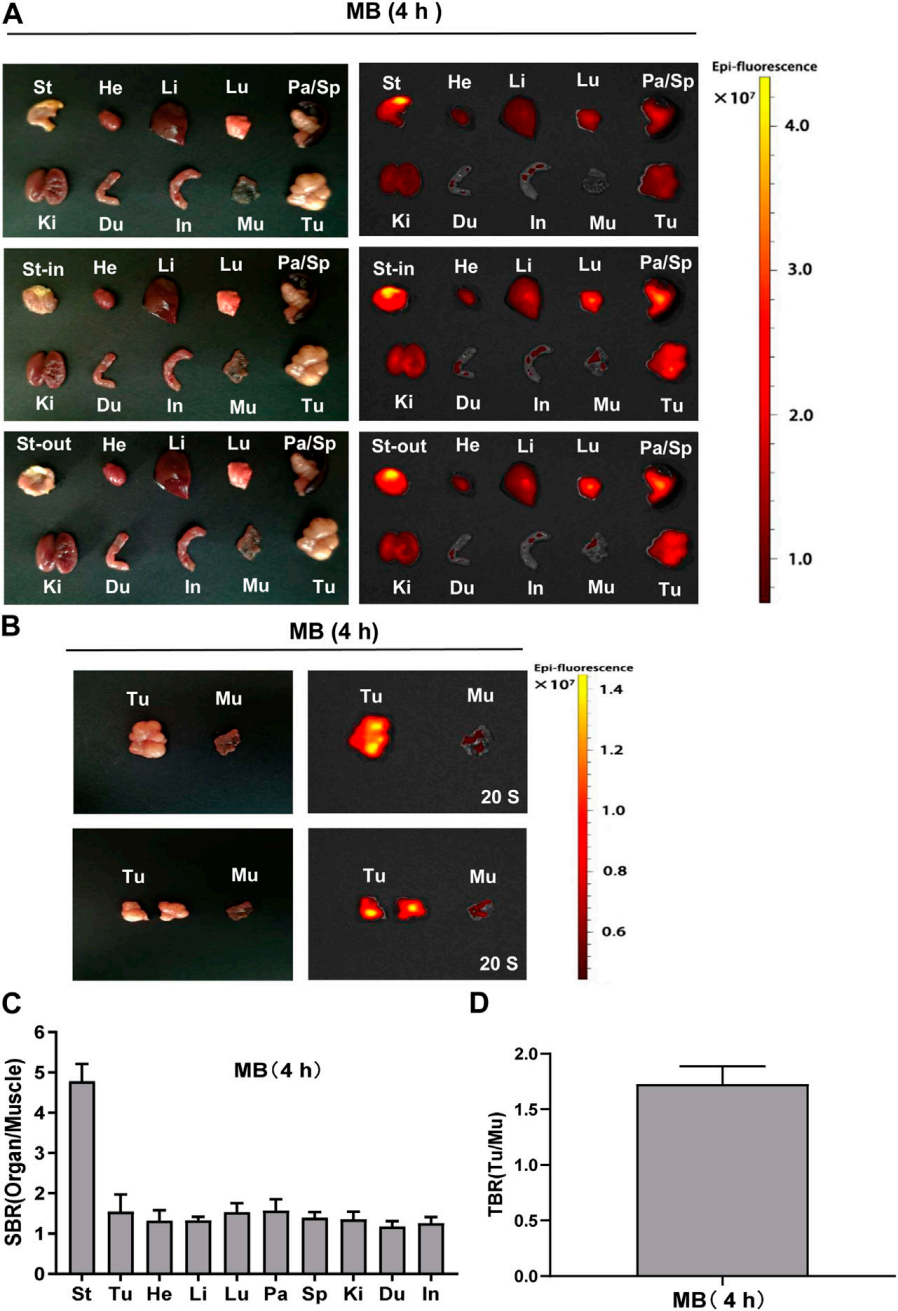
Organ and tumor specificity of MB at 4 h post-injection. MB was injected intravenously into subcutaneous gastric tumor-bearing nude mice, and the NIR fluorescence signals were observed at 4 h post-injection under the NIR imaging system. **(A)** NIR imaging of resected major organs and tumor at 4 h post-injection of MB. **(B)** NIR imaging of resected tumor at 4 h post-injection of MB. **(C)** SBR of each organ against muscle. SBR was calculated by comparing the signals of major organs against the surrounding muscle. **(D)**Tumor-to-background ratio (TBR) of resected tumors. TBR was calculated by comparing the signals of the tumor against the surrounding muscle. Abbreviations are Du, duodenum; He, Heart; In, intestine; Ki, kidneys; Li, liver; Lu, lungs; Mu, muscle; Pa, pancreas; Sp, spleen; St, stomach; St-in, stomach-in; St-out, stomach-out; Tu, tumor. Each sample used three mice. Error bars show mean ± SEM.

Following the intraoperative NIR imaging findings, fluorescence signals of the resected stomach at 4 h post-injection tended to be extraordinarily higher than that of the resected tumor and other organs. To investigate whether the stomach in nude mice could be directly targeted by MB, we cut the stomach and washed it with PBS. The fluorescence signals were still obviously displayed in the stomach-in and stomach-out in subcutaneous gastric tumor-bearing nude mice, suggesting that MB could efficiently target the stomach (Figure 6A). Moreover, fluorescence signals from the resected tumor at 4 h post-injection demonstrated a much higher rate than that of the muscle. After the tumor was cut open, fluorescence signals remained more elevated than the muscle’s (Figure 6B). Consistently, the SBR rate of the resected organs appeared to be significantly high in the stomach (Figure 6C). In addition, the tumor-to-background ratio (TBR) was weakly increased in the tumor at 4 h post-injection of MB (Figure 6D).

**FIGURE 7:**
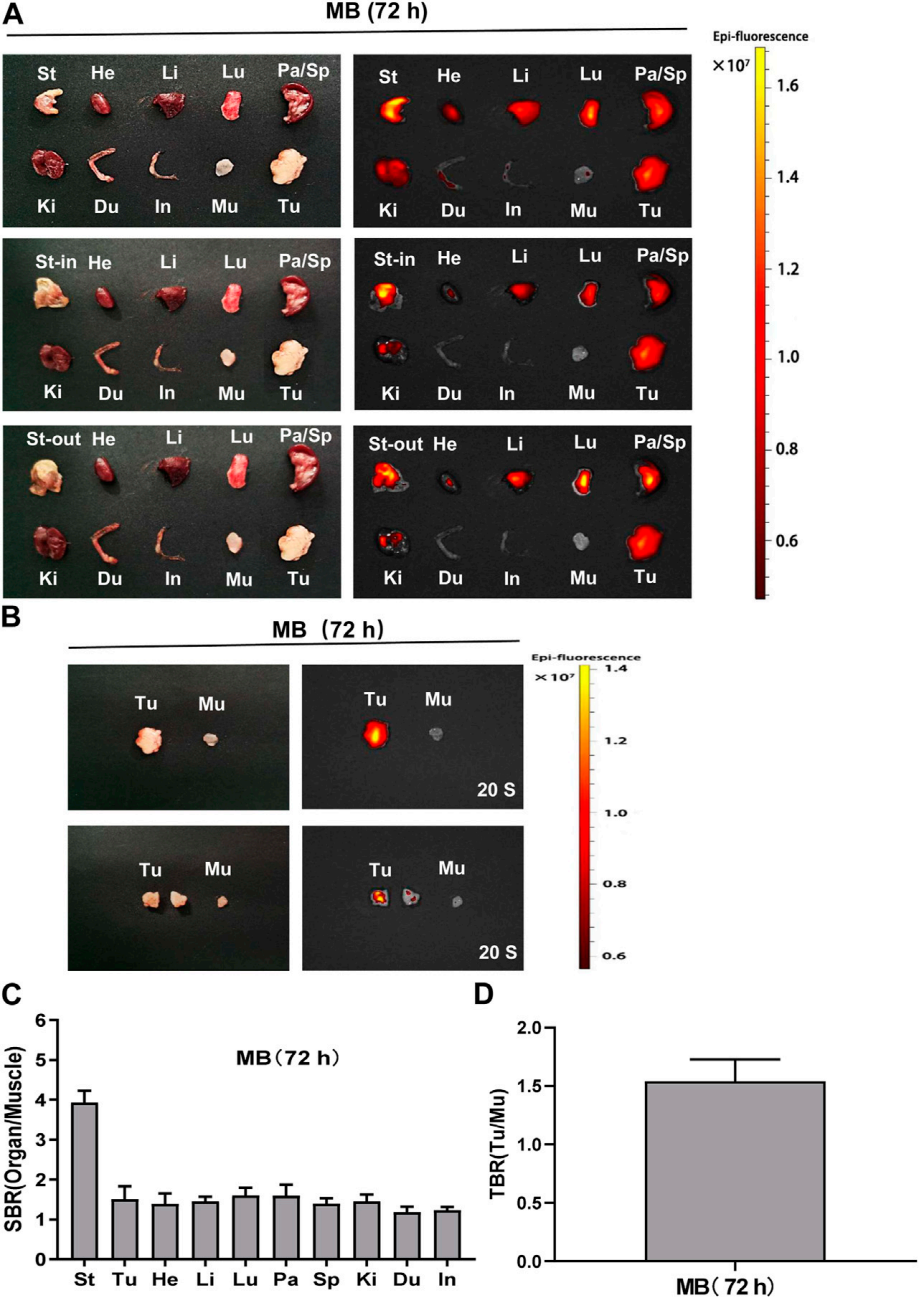
Organ and tumor specificity of MB at 72 h post-injection. MB was injected intravenously into subcutaneous gastric tumor-bearing nude mice, and the NIR fluorescence signals were observed at 72 h post-injection under the NIR imaging system. **(A)** NIR imaging of resected major organs and tumor at 72 h post-injection of MB. **(B)** NIR imaging of resected tumor at 72 h post-injection of MB. **(C)** SBR of each organ against muscle. SBR was calculated by comparing the signals of major organs against the surrounding muscle. **(D)** TBR of resected tumors. TBR was calculated by comparing the signals of the tumor against the surrounding muscle. Abbreviations are Du, duodenum; He, Heart; In, intestine; Ki, kidneys; Li, liver; Lu, lungs; Mu, muscle; Pa, pancreas; Sp, spleen; St, stomach; St-in, stomach-in; St-out, stomach-out; Tu, tumor. Each sample used three mice. Error bars show mean ± SEM.

Consistent with the above findings, fluorescence signals from the resected stomach at 72 h post-injection appeared much higher than that of the resected tumor and other organs. In the stomach-in and stomach-out, the fluorescence was found at 72 h post-injection in subcutaneous gastric tumor-bearing nude mice after the stomach was cut and washed with PBS (Figure 7A). Moreover, fluorescence signals from the resected tumor at 72 h post-injection showed a higher tendency than that of the muscle. After the tumor was cut open, fluorescence signals were mildly higher than the muscle’s (Figure 7B). Consistently, the SBR ratio of the resected organs appeared to be significantly elevated in the stomach (Figure 7C). TBR was weakly increased in the tumor at 72 h post-injection of MB (Figure 7D). It seems that MB can target the stomach, not the subcutaneous gastric tumor.

### 3.6 Real-time NIR targeted imaging in the orthotopic gastric tumor

Although the subcutaneous gastric tumor of nude mice displayed no fluorescence signals after the injection of MB, whether MB can induce efficient tumor-targeted imaging needed to be explored in the orthotopic gastric tumor-bearing nude mice.

**FIGURE 8:**
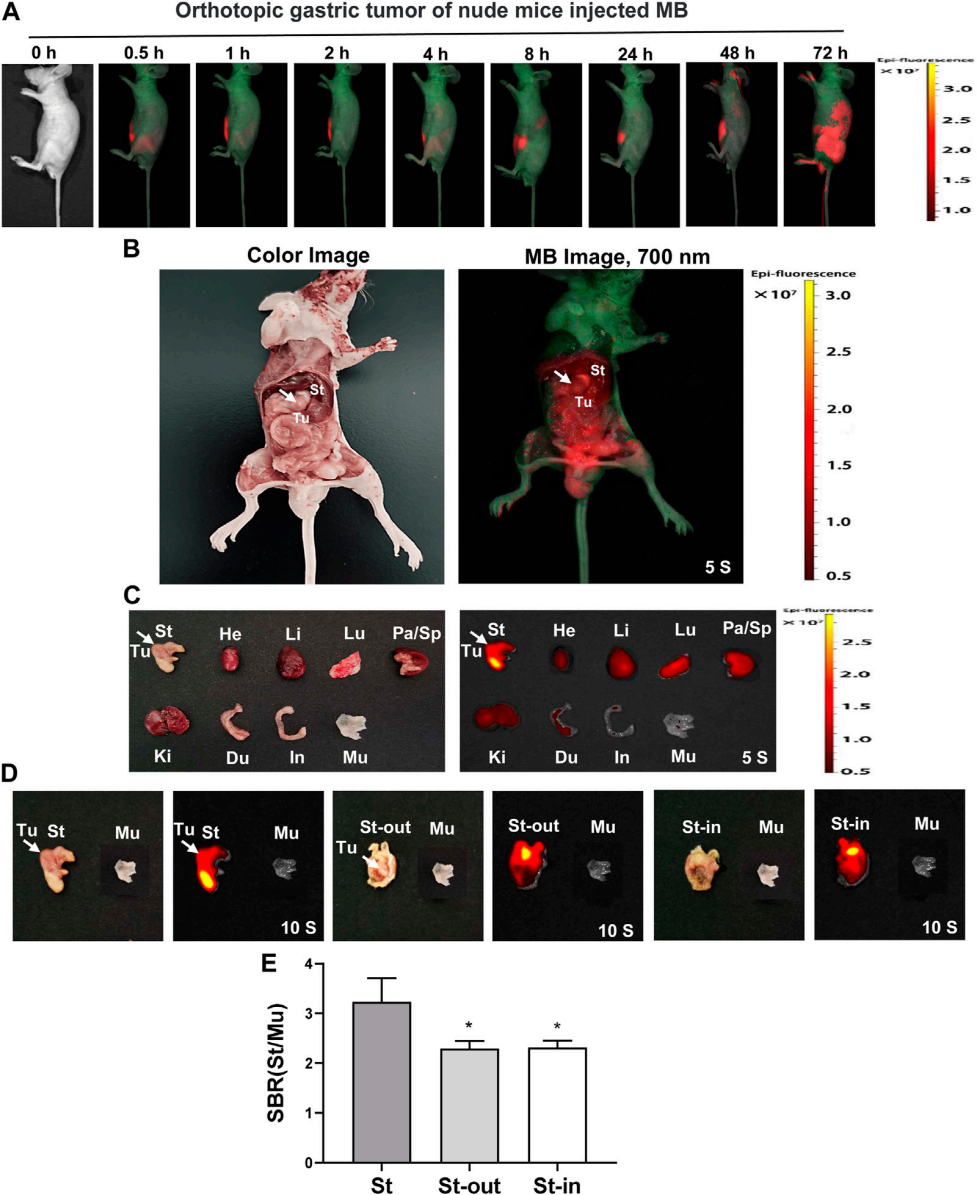
Real-time NIR targeted imaging after the injection of NIR fluorophores MB in the orthotopic gastric tumor of nude mice. MB was injected intravenously into orthotopic gastric tumor-bearing nude mice, and the NIR fluorescence signals were observed at 0.5, 1, 2, 4, 8, 24, 48, and 72 h post-injection under the NIR imaging system. **(A)** Real-time NIR fluorescence imaging of gastric orthotopic tumor-bearing nude mice at different time points. **(B)** NIR imaging of the abdominal cavity of nude mice at 72 h post-injection MB. **(C)** NIR imaging of resected major organs at 72 h post-injection of MB. **(D)** NIR imaging of resected tumor at 72 h post-injection of MB. **(E)** SBR of each organ against muscle. SBR was calculated by comparing the signals of major organs against the surrounding muscle. White arrows indicate tumor grafts in the left flank. Abbreviations are Du, duodenum; He, Heart; In, intestine; Ki, kidneys; Li, liver; Lu, lungs; Mu, muscle; Pa, pancreas; Sp, spleen; St, stomach; St-in, stomach-in; St-out, stomach-out; Tu, tumor. Each sample used three mice. Error bars show mean ± SEM. *p < 0.05 vs. St.

As described in Figure 8A, real-time intraoperative NIR imaging was shown in the orthotopic gastric tumor-bearing nude mice from 0 h to 72 h post-injection of MB.

The stomach in the abdominal cavity of nude mice exhibited distinct signals at 72 h post-injection of MB, but the orthotopic gastric tumor showed no significant signals at 72 h post-injection of MB (Figure 8B). In line with the findings of the intraoperative NIR imaging, the fluorescence signals from the resected stomach at 72 h post-injection displayed a dramatic ascend compared to that of the tumor and other organs. Unfortunately, we cannot find significant NIR fluorescence signals in the orthotopic gastric tumor (Figure 8C). The fluorescence signals of the resected stomach at 72 h post-injection showed higher than that of the muscle. Additionally, the fluorescence signals still appeared in the stomach-in and stomach-out at 72 h post-injection in gastric orthotopic tumor-bearing nude mice after the stomach was dissected and cleaned with PBS. However, the fluorescence signals failed to appear in the orthotopic gastric tumor (Figure 8D). The SBR ratio of the stomach appeared to be distinctly higher than stomach-in and stomach-out (Figure 8E). Thus, it seems suggested that MB can target the stomach, not the orthotopic gastric tumor.

### 3.7 Fluorescence imaging and pathological analysis of MB

**FIGURE 9:**
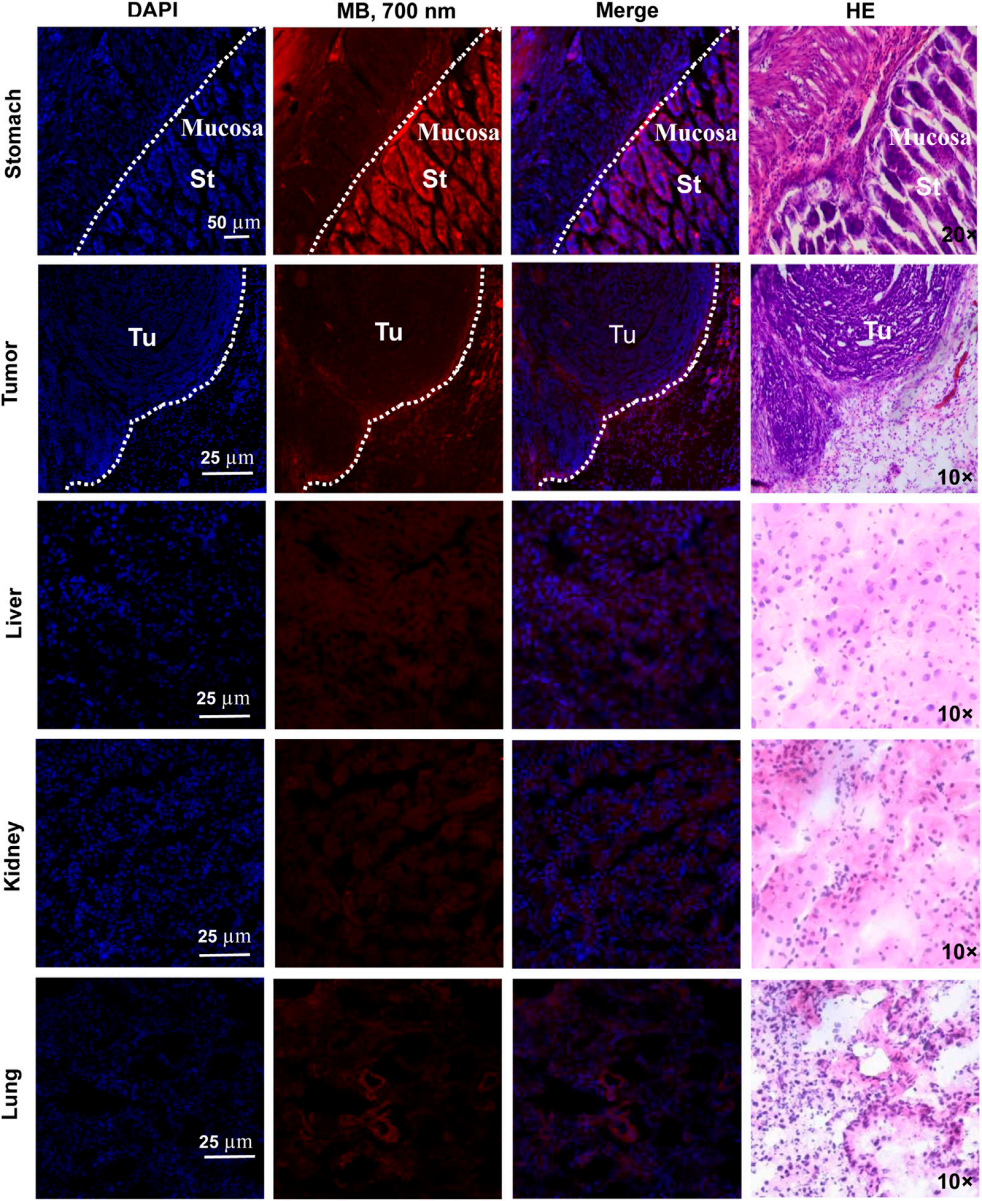
Fluorescence imaging and pathological analysis of MB. MB was injected intravenously into subcutaneous gastric tumor-bearing nude mice 72 h before resection. Then NIR fluorescence and HE staining of the organs (stomach, tumor, liver, kidney, and lung) were imaged under the confocal microscope and Nikon 50i microscope. Abbreviations are St, stomach; Tu, tumor.

It is essential to confirm if the tissue or organ can be damaged by the NIR fluorophores MB. For this reason, we conducted the pathological examination on the main organs (stomach, liver, kidney, and lung) and tumors at 72 h post-injection of MB and did not discover prominent tissues and cellular damages in the nude mice (Figure 9). Meanwhile, no pathological changes were disclosed in the stomach, liver, kidney, or lung after fluorescence examination of the tissue sections. However, compared with other organs (liver, kidney, and lung) and tumors of the nude mice treated with MB, the stomach showed prominent fluorescence, firmly supporting that MB has no inherent specificity for the gastric tumors but specificity for the gastric tissues. Thus, the fluorescence staining of the liver, kidney, lung and tumor tissues with MB was non-specific, and the fluorescence signals were shallow. However, the fluorescence signals of the gastric tissues with MB were remarkable. Moreover, the fluorescence staining of the stomach tissues was mainly located in gastric mucosa, especially the nucleus and cytoplasm possessed significant fluorescence signals.

According to the above results, MB can assist with intraoperative navigation using NIR fluorescence laparoscopy. Therefore, MB will be ideal fluorophores for fluorescence image-guided gastrointestinal surgery. NIR fluorescence with MB is a promising method for potential intraoperative visualization of tissues and structures (Jo and Hyun, 2017). MB has several remarkable advantages in clinical practice and surgical settings, such as its easy, safety, visualization, and intraoperative imaging (Liu et al., 2008). However, MB has some disadvantages, such as low fluorescence efficiency, non-specific uptake, and short retention time (Géczi et al., 2022). Furthermore, MB can adversely affect anaphylaxis, hemolysis, pulse oximetry, and skin and urine discoloration in G6PD-deficient patients (Lwin et al., 2020). Surprisingly, the present study demonstrates that MB has no inherent specificity for the gastric tumor but specificity for the gastric tissues. By taking advantage of the optical properties of MB, a new approach to negative NIR imaging can be performed in gastric tumors. Meanwhile, MB can be used as positive NIR imaging in gastric tissues. Thus, MB-positive and negative NIR imaging are meaningful in targeting gastric tissues and tumors. However, the mechanisms underlying these findings have largely remained elusive. Previous studies reveal that MB can increase gastric acid secretion by activating the H^+^/K^+^ ATPase (Shah et al., 2006). The mechanisms by which MB accumulates and targets the gastric tissues are possibly associated with the H^+^/K^+^ ATPase, and further studies are needed to obtain sufficient evidence to determine the specific mechanisms of MB in stomach diseases.

## 4 Conclusion

In summary, NIR fluorophores MB can exhibit specific uptake by the gastric epithelial and cancer cells. It is primarily found that MB can directly target the stomach in mice. Interestingly, MB was applied for the NIR imaging of gastric cancer cell xenografts, suggesting that MB cannot specifically target subcutaneous and orthotopic gastric tumors in xenograft models. Therefore, it can be concluded that MB has no inherent specificity for gastric tumors but specificity for gastric tissues. Apparently, MB-positive and negative NIR imaging are meaningful in targeting gastric tissues and tumors. MB is expected to represent a helpful NIR agent to secure precise resection margins during the gastrectomy and resection of gastric tumors.

## Data Availability

The original contributions presented in the study are included in the article/supplementary material, further inquiries can be directed to the corresponding author.
